# The Recognition of Hypothalamo-Neurohypophysial Functions by Developing T Cells

**DOI:** 10.1155/1992/98671

**Published:** 1992

**Authors:** F. R. Robert, H. Martens, N. Cormann, A. Benhida, J. Schoenen, V. Geenen

**Affiliations:** 1 Laboratory of Radioimmunology Neuroendocrine-Immunology Unit CHU-B23 University of Liège-Sart Tilman Liège B-4000 Belgium; 2 Laboratory of Pathological Neuroanatomy CHU-B23 University of Liège-Sart Tilman Liège B-4000 Belgium

**Keywords:** Oxytocin, vasopressin, thymic epithelium, neurohypophysial peptide receptors, self-recognition, cryptocrine signaling

## Abstract

Neuropeptide signals and specific neuropeptide receptors have been described in the thymus supporting the concept of a close dialogue between the neuroendocrine and the immune systems at the level of early T-cell differentiation. In this paper, we review recent data about neurohypophysial (NHP)-related peptides detected in the thymus from different species. We suggest that we are dealing in fact with other member(s) of the NHP hormone family, which seems to exert its activity locally through a novel model of cell-to-cell signaling, that of cryptocrine communication. This model involves exchange of signals between thymic epithelial cells and developing thymocytes. The NHP-related peptides have been shown to trigger thymocyte proliferation and could induce immune tolerance of this highly conserved neuroendocrine family.


*Developmental Immunology*, 1992, Vol. 2, pp. 131–140
Reprints available directly from the publisher
Photocopying permitted by license only

© 1992 Harwood Academic Publishers GmbH
Printed in the United Kingdom

### The Recognition of Hypothalamo-Neurohypophysial Functions by Developing T Cells

F. R. ROBERT*, H. MARTENS, N. CORMANN, A. BENHIDA, J. SCHOENEN†, and V. GEENEN

*Laboratory of Radioimmunology, Neuroendocrine-Immunology Unit† and Laboratory of Pathological Neuroanatomy, CHU-B23, University of Liège-Sart Tilman, B-4000 Liège, Belgium*

Neuropeptide signals and specific neuropeptide receptors have been described in the thymus supporting the concept of a close dialogue between the neuroendocrine and the immune systems at the level of early T-cell differentiation. In this paper, we review recent data about neurohypophysial (NHP)-related peptides detected in the thymus from different species. We suggest that we are dealing in fact with other member(s) of the NHP hormone family, which seems to exert its activity locally through a novel model of cell-to-cell signaling, that of cryptocrine communication. This model involves exchange of signals between thymic epithelial cells and developing thymocytes. The NHP-related peptides have been shown to trigger thymocyte proliferation and could induce immune tolerance of this highly conserved neuroendocrine family.

KEYWORDS: Oxytocin, vasopressin, thymic epithelium, neurohypophysial peptide receptors, self-recognition, cryptocrine signaling.

### INTRODUCTION

In the last decade, a number of studies has been reported indicating that various neuropeptides could be found in the thymus or in thymic neoplasms. Thus, neurotensin (NT) (Sundler et al., 1978; Herbst et al., 1987), somatostatin (SS) (Sundler et al., 1978; Geppetti et al., 1987; Fuller and Verity, 1989; Gomariz et al., 1990), oxytocin (OT) (Geenen et al., 1986; Ervin et al., 1988; Argiolas et al., 1990a; Jevremovic et al., 1990), vasopressin (VP) (Markwick et al., 1986; Geenen et al., 1987; Giraud et al., 1990), neurophysins (NPs) (Geenen et al., 1986), tachykinins (TKs) (Geppetti et al., 1988; Weihe et al., 1989; Ericsson et al., 1990; Lorton et al., 1990; Piantelli et al., 1990), neuropeptide Y (NPY) (D'Andrea et al., 1989; Weihe et al., 1989; Ericsson et al., 1990), vasoactive intestinal peptide (VIP) (Felten et al., 1985; Gomariz et al., 1990; Al-Shawaf et al., 1991), calcitonin gene-related peptide (CGRP) (Geppetti et al., 1989; Weihe et al., 1989), opioid peptides (Von Gaudecker et al., 1986; Piantelli et al., 1990), corticotropin (ACTH) (Herbst et al., 1987), cholecystokinin (CCK) (Herbst et al., 1987), and atrial natriuretic peptide (ANP) (Vollmar and Schulz, 1990) have been immunologically, biologically, biochemically, and/or molecularly evidenced. These neuropeptides were not systematically located in the thymic structures. However, some immunoreactive (ir-) neuropeptides were localized in nerve fibers: substance P (SP) (Geppetti et al., 1987, 1988; Weihe et al., 1989; Lorton et al., 1990), CGRP (Weihe et al., 1989), VIP (Felten et al., 1985; Al-Shawaf et al., 1991), NPY (D'Andrea et al., 1989; Weihe et al., 1989). On the other hand, it was suggested that ANP could be localized in thymocytes (Vollmar and Schulz, 1990). Furthermore, NT and SS immunoreactivities were found in sparse stromal cells (Sundler et al., 1978), whereas neurokinin A (NKA) (Ericsson et al., 1990), opioid peptides (Von Gaudecker et al., 1986; Piantelli et al., 1990), OT, VP, and NPs (Robert et al., 1991) were localized in thymic epithelial cells (TEC).

It is assumed that the action of neuropeptides is mediated through specific receptors on the target cells. The presence of such neuropeptide receptors in the thymus also was reported. Thus, binding sites for SP (Shigematsu et al., 1986), ANP (Kurihara et al., 1987), OT (Elands et al., 1988a), and VP (Geenen et al., 1988b) have been reported. Thymic binding sites for SP were found

*Corresponding author.

associated with the vasculature in the medulla, where they may control blood flow and vascular permeability (Shigematsu et al., 1986). On the other hand, specific binding sites for ANP were identified on the rat cortical and medullary thymocytes (Kurihara et al., 1987). It was suggested that they could promote the proliferation and the maturation of thymocytes. Furthermore, it is likely that LH-RH and NKA binding sites are localized on thymocytes because they exert a mitogenic effect on these cells, although it is not excluded that this effect could be mediated by other cell types present in cell cultures (Marchetti et al., 1989; Söder and Hellström, 1989).

In this review, we reconsider the observations about neurohypophysial (NHP)-related peptide signals (OT, VP, and associated NPs) as well as their intrathymic receptors in the context of a new concept of intercellular dialogue, the cryptocrine communication, which was recently proposed for testicular Sertoli cells and thymic nurse cells (TNC) (Funder, 1990). Briefly, cryptocrine signaling differs from the paracrine one in that it involves exchange of signals between specialized epithelial cells (Sertoli in the testis, TNC or other TEC in the thymus) and migratory developing elements (spermatids and thymocytes, respectively).

### THE NEUROHYPOPHYSIAL PEPTIDE FAMILY

Ten NHP hormones, naturally occurring in vertebrates, constitute a family of nonapeptides, highly conserved throughout evolution (Acher and Chauvet, 1988). They can be arranged in two lineages corresponding to the OT-like and VP-like hormones. These peptides all consist of nine amino acids with cysteine residues in positions one and six forming a disulfide bridge. Substitutions occur at positions 4 and 8 and, less frequently, at positions 2 and 3 (cf. Table 1). Vasotocin (VT), originally a synthetical hybrid of oxytocin ring and Arg-vasopressin side chain, was later found in the brain of all the adult nonmammalian vertebrates. It appears to be the most primitive of the NHP peptides and may be the common ancestor of all other NHP hormones. However, this molecule has also been detected and characterized in fetal mammals (Pavel, 1975; Smith and McIntosh, 1983; Ervin et al., 1985) as

TABLE 1
Neurohypophysial Hormone Family: Nonapeptides Naturally Occurring in Vertebrates

| Oxytocin Lineage | | | | | | | | | |
|---|---|---|---|---|---|---|---|---|---|
| 1 | 2 | 3 | 4 | 5 | 6 | 7 | 8 | 9 | |
| Cys | Tyr | Ile | Gln | Asn | Cys | Pro | Leu | Gly-NH2 | Oxytocin (placental mammals) |
| Cys | Tyr | Ile | Gln | Asn | Cys | Pro | **Ile** | Gly-NH2 | Mesotocin (marsupials, birds, reptiles, amphibians, lungfishes) |
| Cys | Tyr | Ile | **Ser** | Asn | Cys | Pro | **Ile** | Gly-NH2 | Isotocin (bony fishes) |
| Cys | Tyr | Ile | **Ser** | Asn | Cys | Pro | **Gln** | Gly-NH2 | Glumitocin (rays) |
| Cys | Tyr | Ile | Gln | Asn | Cys | Pro | **Val** | Gly-NH2 | Valitocin (sharks) |
| Cys | Tyr | Ile | **Asn** | Asn | Cys | Pro | Leu | Gly-NH2 | Aspargtocin (sharks) |
| **Vasopressin Lineage** | | | | | | | | | |
| 1 | 2 | 3 | 4 | 5 | 6 | 7 | 8 | 9 | |
| Cys | Tyr | Phe | Gln | Asn | Cys | Pro | Arg | Gly-NH2 | Arg-Vasopressin (mammals) |
| Cys | Tyr | Phe | Gln | Asn | Cys | Pro | **Lys** | Gly-NH2 | Lys-Vasopressin (pig, macropodids) |
| Cys | **Phe** | Phe | Gln | Asn | Cys | Pro | Arg | Gly-NH2 | Phenypressin (macropodids) |
| Cys | Tyr | **Ile** | Gln | Asn | Cys | Pro | Arg | Gly-NH2 | Vasotocin (non-mammalian vertebrates) |

Aminoacid substitutions in the lineage are indicated in bold characters.

well as in the mammalian pineal gland (Pavel et al., 1975; Legros et al., 1976; Nieuwenhuis, 1984).

Most vertebrates usually produce two NHP peptides: an OT-like hormone, involved in reproductive processes (parturition and lactation), and a VP-like peptide, engaged in the regulation of water metabolism. However, a single nonapeptide, VT, is found in the lamprey, one of the most primitive vertebrates (Acher and Chauvet, 1988). Therefore, it has been assumed that the OT-like and the VP-like lineages arose from the duplication of one ancestral gene. Recent observations have further supported this postulated molecular mechanism (Morley et al., 1990).

In mammals, the neurohormones of OT and VP are under the control of two independent genes expressed in magnocellular neurons of the hypothalamus. They are synthesized as large molecular weight precusors, which are then cleaved during axonal transport (Fig. 1). Prooxyphysin (Pro-OT) is a 16-kD protein containing the OT sequence at its N-terminus, which is separated from the OT-associated neurophysin (OT-NP) by a Gly-Lys-Arg spacer sequence, and which contains an extra C-terminal amino acid (Land et al., 1983). Propressophysin (Pro-VP) is a 20-kD glycoprotein that contains the VP sequence at the N-terminus separated from its associated NP by the same tripeptide spacer (Gly-Lys-Arg). In addition, Pro-VP contains a 39 amino acid terminal glycopeptide, the copeptin, which is separated from VP-NP by a single Arg spacer (Land et al., 1982).

Neurophysins are small (93–95 residues) acidic proteins. The central part (residues 10–74), which is encoded by the second exon, is notably conserved during the evolutionary history of these peptides (Ivell and Richter, 1984). This sequence is nearly identical between the two NPs of a given species and between various animal species. Variations between lineages occur in the N-terminal part (residues 1–9) and in the C-terminal part (residues 76–95) of the NP molecule. Neurophysins exert a role of carrier for VP and OT in the NHP system, but their high degree of conservation throughout evolution suggests other physiological roles that remain to be established.

On the basis of both functional and pharmacological criteria, two types of VP receptors can be distinguished: $V_1$ receptors that mediate pressor and glycogenic responses to VP, by activating

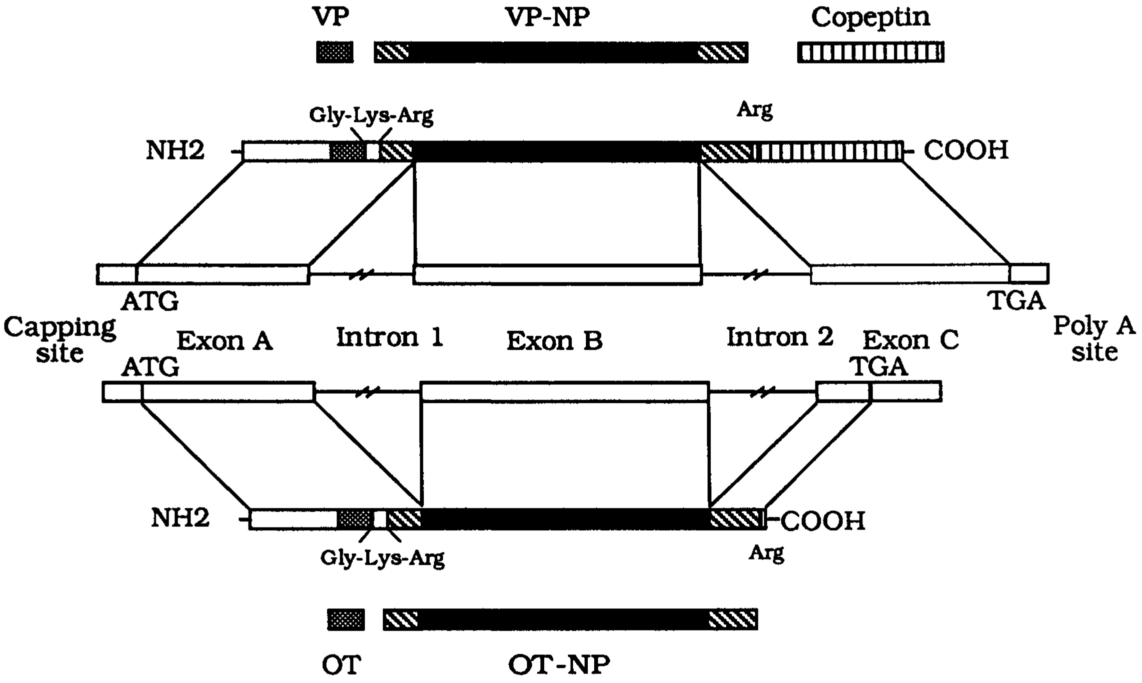

FIGURE 1. Structure of oxytocin and vasopressin genes expressed in the hypothalamus (redrawn from Richter and Schmale, 1984).

phosphoinositide breakdown and elevating the concentration of intracellular calcium, and $V_2$ renal receptors, leading to the activation of adenylate cyclase (Michell et al., 1979). Further, pharmacological studies using peptide binding assays have led to distinguish two $V_1$ receptors subtypes named $V_{1a}$ and $V_{1b}$ (Jard et al., 1986).

OT receptors were described in the uterus, mammary gland, oviduct, and hippocampus. These receptors seem to be very closely related to the $V_1$ type and also are coupled to phosphoinositide metabolism (Flint et al., 1986).

### EVIDENCE FOR NEUROHYPOPHYSIAL-LIKE PEPTIDES IN THYMIC EXTRACTS

We have previously demonstrated that ir-OT could be extracted by acetic acid from human thymuses as evidenced by specific radioimmunoassay (RIA) using antiserum $AsO_2$ (Geenen et al., 1986). On isolated rat uterus, thymic extracts induced oxytocic contraction and the quantitative bioactivity was in agreement with the amounts detected by RIA. Ir-NPs also were detected in human thymic extracts using the antiserum A/5/IV. The molar ratio of ir-OT (2.2–18.4 ng/g) to ir-NPs (24–142 ng/g) was similar to that found in the hypothalamus (1:1), suggesting a local synthesis by cleavage from a common precursor. Ir-VP and ir-VP-NP also were detected in human thymic extracts (0.01–0.06 ng/g and 34–90 ng/g, respectively). The further finding of positive dot blot hybridizations of human thymic mRNA with bovine OT and VP cDNA probes constituted another argument for in situ synthesis, although this observation does not establish a conclusive demonstration of intrathymic OT and VP gene expression (Geenen et al., 1987).

Thymic OT was further characterized by gel filtration on G-75 Sephadex: separate peaks of ir-OT and ir-NP were evidenced at the same positions as their respective reference preparations. HPLC analysis of thymus extracts showed a single peak of ir-OT with a similar elution site as synthetic OT (Geenen et al., 1986).

Independently, ir-VP has been described in thymic extracts of rats and mice (Markwick et al., 1986), and OT-, VP- and VT-like peptides were reported in ovine thymus (Ervin et al., 1988). The presence of ir-OT was recently confirmed in fetal human thymus (Jevremovic et al., 1990) and in rat thymus (Argiolas et al., 1990a). The finding of a modulation of rat thymic ir-OT concentrations after diverse manipulations further supports a local intrathymic synthesis of an OT-like peptide (Argiolas et al., 1990b).

### LOCALIZATION OF NEUROHYPOPHYSIAL-LIKE PEPTIDES IN HUMAN THYMUS

By using polyclonal antibodies, we localized ir-OT and ir-VP in the subcapsular cortex (SCC) and in the medulla of human thymus, as well as in mouse lymphoepithelial complexes called thymic nurse cells (TNC) (Wekerle and Ketelsen, 1980; Geenen et al., 1988a). Furthermore, we confirmed the presence of ir-OT in human, rat, and mouse thymuses with O33, a monoclonal antibody (Mab) directed to OT specifically recognizing hypothalamic magnocellular neurons (Fig. 2A). We only observed a labeling of the stromal component of thymuses; thymocytes and nerve fibers were never visualized. In human thymus, O33 revealed two immunoreactive areas (Fig. 2B): a monolayer of flattened cells in the SCC and a dense reticular network of stellate cells in the medulla, whereas in the inner cortex, very few scattered stellate cells were labeled. In the mouse thymus, this antibody mainly labels a homogeneous network of cortical reticular cells and some medullary stromal cells (Fig. 2C). Several strains of mice (C3H, BALB/c, C57BL/Ka, CBA, SJL/J) as well as Wistar rats (Fig. 2D) exhibited the same pattern of reactivity. Moreover, the thymus of Brattleboro rats, which exhibit a deficit of the hypothalamic VP gene caused by a single deletion (Richter and Schmale, 1984), also was stained by O33 (Fig. 2E).

Using double immunofluorescence staining with A/5/III, a rabbit serum anti-NPs, we found that the same cells express together ir-OT and ir-NP (Robert et al., 1991). Among the Mabs to VP, we found that BER-312 was the brightest though it labeled thymus sections less intensely than O33, in accordance with the lower concentration of ir-VP found in human thymuses. The pattern of reactivity of BER-312 on human thymus was similar to that obtained with O33 (Robert et al., 1991).

Further, in human thymus, we showed that the stromal cells containing ir-neuropeptides correspond to a subset of epithelial cells expressing KL1- and KL4-defined cytokeratins (Robert et al., 1991). The epithelial nature of the cells containing NHP-related peptides was confirmed, in rodent thymuses, using an antiserum to human keratins and KL4 (data not shown). However, KL1 only stained a few medullary TEC in rodents (Nicolas et al., 1989), whereas it strongly labels cortical and medullary TEC from humans (Robert et al., 1991), evidencing a certain disparity between rodent and human TEC.

Besides, both in humans and rodents, it

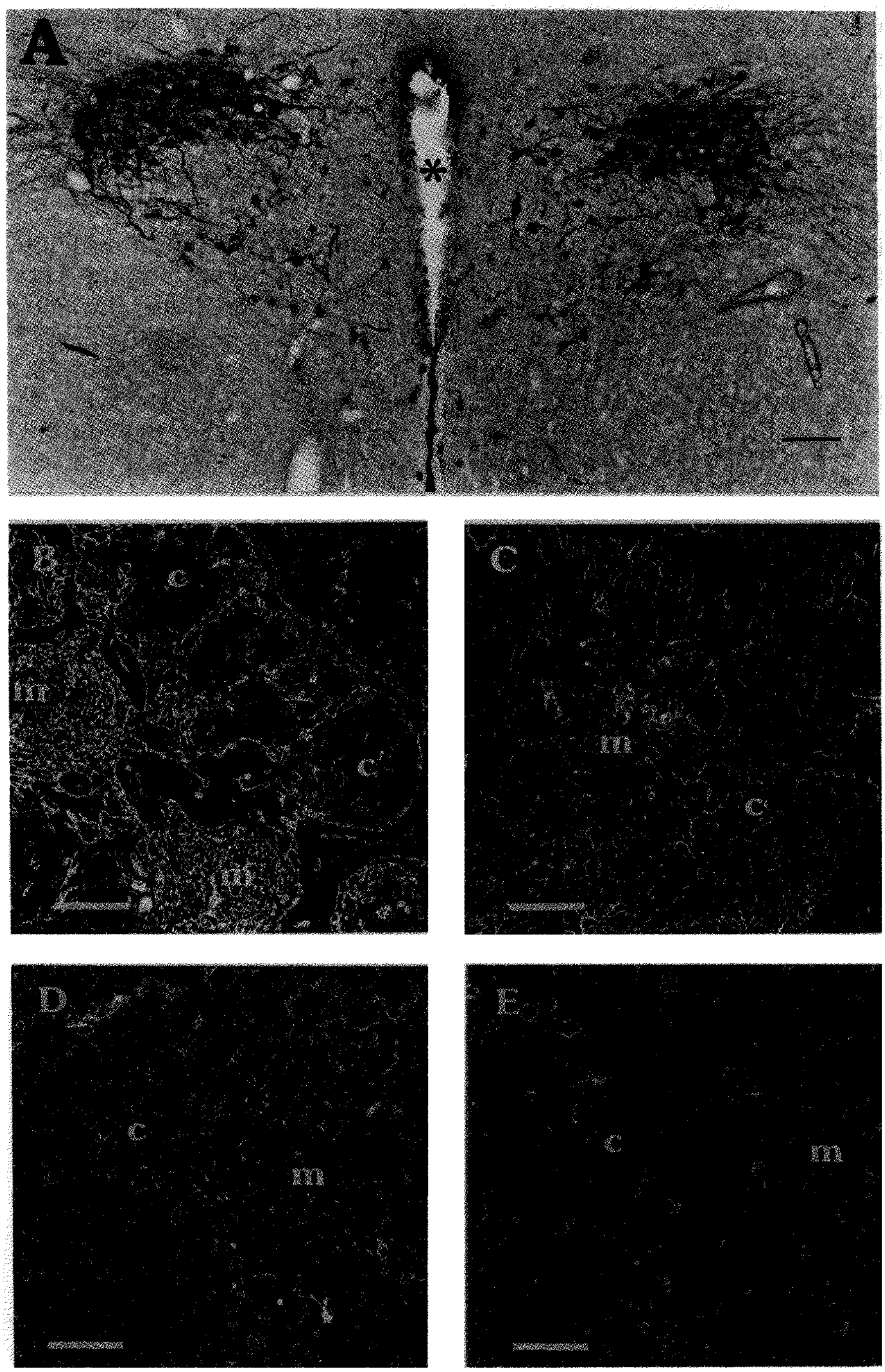

FIGURE 2. Immunostaining with a Mab to oxytocin (O33). (A) Transverse section (25 $\mu$m thick) through the hypothalamus of a 3-month-old female Wistar rat. It was immunostained by successive incubation with (1) Mab O33 (1:2000); (2) goat antimouse IgM conjugated with biotin (1:800); (3) avidin-peroxidase (1:200); (4) 3,3'-diaminobenzidine tetrahydrochloride (for detailed protocol, see Burgeon et al., 1991). In the paraventricular nucleus, which contains oxytocin and vasopressin magnocellular neurons, neuronal cell bodies and dendrites are heavily stained by 033. *:third ventricle. Scale bar: 20 $\mu$m. (B) Three-year-old male human thymus section (5 $\mu$m) labeled with O33 (1:100) and FITC goat antimouse Ig (1/40). The SCC and the medulla are strongly labeled. c: cortex; m: medulla. Scale bar=40 $\mu$m. (C) One-month-old female C3H mouse thymus section (5 $\mu$m) labeled with O33 (1:100) and FITC goat antimouse Ig (1/40). Identical fluorescent staining of both cortical and medullary regions was observed. However, a network of labeled cell processes is visible throughout the cortex, whereas, in the medulla, largest globular cells are stained. c: cortex; m: medulla. Scale bar=20 $\mu$m. (D) One-month-old male Wistar rat thymus section (5 $\mu$m) labeled with O33 (1:100) and FITC goat antimouse Ig (1/40). The staining is similar to that of C3H mouse but slightly weaker. c: cortex; m: medulla. Scale bar=20 $\mu$m. (E) One-month-old female Brattleboro rat thymus section (5 $\mu$m) labeled with O33 (1:100) and FITC goat antimouse Ig (1/40). Positive labeling was observed in the thymus of this mutant rat defective in expression of hypothalamic vasopressin precursor. c: cortex; m: medulla. Scale bar= 20 $\mu$m.

appeared that TEC containing ir-neuropeptides also are labeled by A2B5 (Geenen et al., 1988a; Robert et al., 1991), which recognizes the thymic neuroendocrine microenvironment producing the so-called thymic hormones (Haynes et al., 1983; Savino and Dardenne, 1984). In human thymuses, we found that these TEC also contain some interleukin-1 (IL-1$\beta$) immunoreactivity that was colocalized with ir-OT (Robert et al., 1991). It is as well possible that they produce other lymphokines (interleukin-6, colony-stimulating factors, leukemia inhibitory factor) that were evidenced in culture supernatants of human TEC (Le et al., 1990). Furthermore, we observed that, in the rat thymus, a subset of O33$^+$ TEC also was labeled by a specific antiserum directed to NKA, kindly provided by E. Brodin (Karolinska Institute, Stockholm, Sweden).

### THYMIC NEUROHYPOPHYSIAL PEPTIDE RECEPTORS

Specific OT receptors with a high affinity for OT were demonstrated in the rat thymus with the use of a selective, radioiodinated OT antagonist (Elands et al., 1990). These receptors were detected in membrane preparations and on both mature and immature thymocytes, whereas VP receptors of the $V_1$ type were identified on splenic lymphocytes. Using $^3$H-AVP and $^3$H-OT, we have found specific binding sites on $RL_{12}$-NP, an immature T-cell line derived from a murine X-ray-induced thymic lymphoma (Geenen et al., 1988b), as well as on murine CTL-L, a cytotoxic T-cell line (Martens et al., in press). Some heterogeneity of [$^3$H]-AVP and [$^3$H]-OT binding sites was confirmed by computer analysis (LIGAND program). The highest affinity population (100–1,000 binding sites/cell) displayed a Kd ranging between 0.15 and 0.1 nM, depending on the cell line. Displacement curves obtained with different OT and VP analogs indicated that the receptors were of the OT and/or the VP $V_1$ type. The functional transduction capacity of these receptors was demonstrated, on RL12-NP and CTL-L cells, by the ability of different members of the NHP peptide family (VP, OT, and VT) to induce a breakdown of membrane phosphoinositides together with an increase of cytoplasmic inositol-triphosphate (Martens et al., in press). The heterogeneity of the receptor expressed by T cells was further evidenced by the inhibition of transduction in RL12-NP cells by a $V_1$ antagonist, while this inhibition was observed in CTL-L by both OT and V1 antagonists. As a consequence, these observations establish the expression and the functionality of NHP peptide receptors on immature and differentiated T cells. Furthermore, we have observed that OT, VP, and VT increase [$^3$H]-TdR incorporation by human thymocytes cultured in serum-free medium (Martens et al., in press).

### IDENTITY OF THYMIC ir-OT AND ir-VP

Altogether, these data establish the intrathymic synthesis of peptide signal(s) exhibiting very close analogy with NHP peptides and the expression of functional cognate receptors by T cells. However, the presence of a material in TEC that exhibits immunological similarities with OT and, to a lesser extent with VP, does not substantiate that we are dealing with authentic OT and VP. Most data rely upon antibodies to OT, VP, and NPs that could, as well, detect some related epitopes. Some molecular differences between hypothalamic and thymic nonapeptides are obvious, since our Mabs all recognized OT or VP in brain slices (Robert et al., 1985; Burgeon et al., 1991), whereas human thymus sections were only brightly stained by O33. As O13 does not stain thymus sections, it could be that the tail part of OT differs in the thymus and in the brain. In addition, the coexistence of ir-OT and ir-VP in TEC provides another conflicting debate. Finally, classical molecular biological methods failed to demonstrate the intrathymic expression of known hypothalamic OT and VP genes. Therefore, thymic ir-OT and ir-VP appear like peptide(s) related to, but distinct, from hypothalamic OT and VP. The expression of this OT/VP-like peptide could be under the control of a different gene coding for another natural NHP peptide, like VT, expressed preferentially during fetal development in peripheral organs. A different splicing of known OT and VP mRNA also can be considered and these questions are under current investigation.

Furthermore, the pharmacological characterization of thymic OT receptors is not selective enough to assume that the binding peptide is authentic OT, as it has been shown that OT receptors from the hippocampus, the uterus and the mammary gland does not discriminate between OT, VP, and VT (Maggi et al., 1987; Elands et al., 1988b).

### PHYSIOLOGICAL IMPLICATIONS

The physiological actions of OT- and VP-like peptides within the thymus remain to be further defined. They are not secreted in vitro (unpublished observation) and the classical model of neurosecretion is therefore questionable with regard to thymic epithelium-derived NHP signals. Rather, they seem implicated in thymic cell-to-cell interactions according to the novel "cryptocrine" model (Geenen et al., 1991a and 1991b). The affinity of T-cell receptors for OT/VP-like peptides (around $10^{-10}$ M, in best conditions) provides another argument in favor of this concept. The biological significance of a binding to lymphocytes of blood VP and OT neurohormones (concentrations around $10^{-12}$ M) would be rather irrelevant in any case.

These ir-neuropeptides are localized in areas (SCC in human thymus, TNC in murine thymus) where thymocytes divide actively. It was reported that VP/OT exert a mitogenic effect on several cell types including rat (Whitfield et al., 1970) and human (Martens et al., in press) thymocytes, as well as rat bone marrow cells (Hunt et al., 1977). On cultured fibroblasts, VP is considered to deliver an early regulatory signal of mitogenesis (Rozengurt, 1986); therefore, one can reasonably consider that thymic VP/OT-related peptide(s) provides accessory activation signals for thymocyte proliferation.

Ir-OT/VP are also detected in the medullary epithelium of thymus from different species and in cortical epithelium from rodent thymuses. Tolerogenic properties have been reported for murine TEC including TNC (Lorenz and Allen, 1989; Marrack et al., 1989; Webb and Sprent, 1990), at least for some antigens (Ransom et al., 1991). Obviously, the tolerance to OT seems particularly powerful and autoimmune processes have never been described against OT or OT-producing neurones. This is not the case for VP because some idiopathic diabetes insipidus seem to be secondary to autoimmune "hypothalamitis" (Scherbaum and Bottazzo, 1983). Another indirect argument for the tolerogenic aspect of OT is derived from published work and from our personal experience. Indeed, the frequency as well as titers of antisera are higher for VP than for OT, and their immunodominant epitope is generally located in the cyclic part of these nonapeptides. Their protection from an autoaggression by the immune system presumes the presentation in the thymus of a peptide (or of a self-immunodominant epitope) representative of this family. The tolerogenic properties of NHP-related peptide(s) and the hypothesis of their intrathymic presentation by major histocompatibility complex proteins are actually investigated in our laboratory.

### MATERIALS AND METHODS

### Antibodies

The antioxytocin serum $AsO_2$ was raised in rabbit against synthetic OT coupled to bovine thyroglobulin and was previously described (Geenen et al., 1985). These antibodies react with OT: 100%, VT: 40%, and VP: 0.3%.

The rabbit serum against arginine-vasopressin used in RIA (AS2) was mainly directed to the C-terminal linear side chain of the VP molecule (Smitz et al., 1988).

Rabbit antisera to bovine NPs (A/5/III, A/5/IV) were previously shown to recognize the central part of the molecule that is common to OT-NP and to VP-NP and is well conserved through mammals (Legros, 1975).

To characterize VP-NP, we used a specific rabbit antiserum that did not recognize OT-NP, neither OT or VP (Legros and Ansseau, 1989).

The characterization of Mabs to oxytocin was published in detail elsewhere (Burgeon et al., 1991). One Mab (O13) is very specific for OT as it discriminates OT from isotocin (IT) or VP either conjugated to ovalbumin or as free peptides. It does not label the suprachiasmatic nucleus (claimed to contain only VP) and is absorbed by OT- but not by VP-coated beads. As its binding to $[^{125}I]$-OT is inhibited by OT but not by analogs differing at position 8, we conclude that the C-terminal linear chain of the OT molecule is included in the epitope recognized by O13. We also characterized two Mabs (O22 and O33) that are not so strictly specific for OT as they recognize not only OT but also IT and VP conjugates.

They label the rat suprachiasmatic nucleus and their immunoreactivity is as well absorbed by OT- as by VP-coated beads. As they do not bind to [$^{125}$I]-OT, we assume that they react with the cyclic part of OT and probably recognize determinant(s) shared by several natural peptides. However, some differences between O22 and O33 were observed: O22 recognizes IT better than VP and it labels nerve fibers more strongly than O13 and O33 (Burgeon et al., 1991). O13 and O22 belong to the $IgG_1$ subclass, whereas O33 is an IgM.

The production and characterization of Mabs to vasopressin were previously reported (Robert et al., 1985). Briefly, seven hybridomas ($IgG_1$) reacting with VP-thyroglobulin complexes but not with thyroglobulin alone were established. All Mabs bound [$^{125}$I]-VP and stained Long Evans but not Brattleboro (a VP defective mutant) rat neurohypophysis. These antibodies seem to be strictly directed to the cyclic moiety of the VP molecule. However, BER-312 and BER-624, but not CLA-223, were able to detect frog NHP peptides (vasotocin and/or mesotocin), suggesting that they recognize a slightly different epitope.

To further characterize stromal cells, we used commercially available Mabs to human cytokeratins (KL1 and KL4; Immunotech, Marseille, France) as well as a rabbit antiserum directed against human cytokeratins (A-575, Dako, Copenhagen, Denmark). Mouse hybridoma A2B5 (ATCC HB-29) was grown in our lab and used as undiluted supernatants. Antibodies to IL-1$\beta$ were produced by Medgenix Diagnostics (Fleurus, Belgium).

### Immunohistochemistry

Thymus fragments were obtained from children undergoing corrective cardiovascular surgery or from 1-month-old mice (C3H, BALB/c, C57BL/Ka, CBA, SJL/J) and rats (Wistar and Brattleboro). These fragments were embedded in Tissue-tek, immediately frozen in dry ice and then stored at −70 °C. Frozen sections (5 $\mu$m) were cut on a cryostat, air dried, and postfixed with picric acid/formaldehyde.

Sections were incubated for 30 min, at room temperature, with 10% normal goat serum. First-step antibodies were incubated overnight at +4 °C (antineuropeptides) or 30 min at room temperature. Fluoresceine- or rhodamine-conjugated goat antisera to mouse IgG or IgM (Fc specific) or to rabbit Ig (Nordic, Tilburg, Netherlands) were used as second-step reagent for 30 min at room temperature. The double-staining procedure involved sequential incubations of tissue sections.

No specific staining was observed in the thymus when dilution buffer, normal rabbit serum (NRS; Dako, Copenhagen, Denmark), or anti-BSA or anti-OVA control ascites ($IgG_1$ or IgM) were used in place of the primary antibodies. Preadsorption of O33 and BER-312 monoclonal antibodies with homologous synthetic peptides, OT and VP, respectively, coupled to CNBr-activated sepharose-4B (Pharmacia, Sweden), abolished the immunostaining. We also checked that our Mabs to neuropeptides did not detect a cross-reacting antigen in epidermal epithelium.

### ACKNOWLEDGMENTS

Françoise Robert is supported by INSERM (France). Vincent Geenen is Research Associate and J. Schoenen is Research Director of FNRS (Belgium). These studies are supported by the Special Fund for Scientific Research of the University of Liège, the Association Française contre les Myopathies, the Belgian Fund for Medical Scientific Research (grants no. 3.4562.90 and 7.4611.31) and the European Science Foundation (Neuroimmunomodulation Network).

*(Received May 14, 1991)*

*(Accepted September 25, 1991)*

### REFERENCES

Acher R., and Chauvet J. (1988). Structure, processing and evolution of the neurohypophysial hormone-neurophysin precursors. Biochimie **70:** 1197–1207.

Al-Shawaf A.A., Kendall M.D., and Cowen T. (1991). Identification of neural profiles containing vasoactive intestinal polypeptide, acetylcholinesterase and catecholamines in the rat thymus. J. Anat. **174:** 131–143.

Argiolas A., Gessa G.L., Melis M.R., Stancampiano R., and Vaccari A. (1990b). Effects of neonatal and adult thyroid dysfunction on thymic oxytocin. Neuroendocrinol. **52:** 556–559.

Argiolas A., Melis M.R., Stancampiano R., Mauri A., and Gessa G.L. (1990a). Hypothalamic modulation of immunoreactive oxytocin in the rat thymus. Peptides **11:** 539–543.

Burgeon E., Chapleur M., Schoenen J., Remichius D., Legros J.J., Geenen V., and Robert F. (1991). Monoclonal antibodies to oxytocin: Production and characterization. J. Neuroimmunol. **31:** 235–244.

D'Andrea V., Artico M., Capuano L., Gallottini L., and Ambrogi V. (1989). Dimostrazione immunoistochimica del neuropeptide Y nel timo umano normale e nel timoma. Medicina (Florence) **9:** 299–301.

Elands J., Barberis C., and Jard S. (1988b). [$^3$H]-[Thr$^4$, Gly$^7$] OT: A highly selective ligand for central and peripheral OT receptors. Am. J. Physiol. **254:** E31–E38.

Elands J., Resink A., and De Kloet E.R. (1988a). Oxytocin receptors in the rat thymic gland. Eur. J. Pharmacol. **151:** 345–346.

Elands J., Resink A., and De Kloet E.R. (1990). Neurohypophyseal hormone receptors in the rat thymus, spleen, and lymphocytes. Endocrinology **126:** 2703–2710.

Ericsson A., Geenen V., Robert F., Legros J.J., Vrindts-Gevaert Y., Franchimont P., Brene S., and Persson H. (1990). Expression of preprotachykinin-A and neuropeptide-Y messenger RNA in the thymus. Molec. Endocrinol. **4:** 1211–1218.

Ervin M.G., Leake R.D., Ross M.G., Calvario G.C., and Fischer D.A. (1985). Arginine vasotocin in ovine fetal blood, urine, and amniotic fluid. J. Clin. Invest. **75:** 1696–1701.

Ervin M.G., Polk D.H., Humme J.A., Ross M.G., Leake R.D. and Fischer D.A. (1988). Immunoreactive vasotocin, vasopressin and oxytocin in ovine fetal and neonatal thymus glands. Clin. Res. **36:** 217A.

Felten D.L., Felten S.Y., Carlson S.L., Olschowska J.A., and Livnat, S. (1985). Noradrenergic and peptidergic innervation of lymphoid tissue. J. Immunol. **135:** 755s–765s.

Flint A.P.F., Leat W.M.F., Sheldrick E.L., and Stewart H.J. (1986). Stimulation of phosphoinositide hydrolysis by oxytocin and the mechanism by which oxytocin controls prostaglandin synthesis in the ovine endometrium. Biochem. J. **237:** 797–805.

Fuller, P.J., and Verity K. (1989). Somatostatin gene expression in the thymus gland. J. Immunol. **143:** 1015–1017.

Funder, J.W. (1990). Paracrine, cryptocrine, acrocrine, Mol. Cell. Endocrinol. **70:** C21–C24.

Geenen V., Defresne, M.P., Legros J.J., Franchimont P., and Boniver J. (1988a). The neurohormonal thymic microenvironment: Immunocytochemical evidence that thymic nurse cells are neuroendocrine cells. Neuroendocrinol. **47:** 365–368.

Geenen V., Legros J.J., Franchimont P., Baudrihaye M., Defresne M.P. and Boniver J. (1986). The neuroendocrine thymus: Coexistence of oxytocin and neurophysin in the human thymus. Science **232:** 508–511.

Geenen V., Legros J.J., Franchimont P., Defresne M.P., Boniver J., Ivell R., and Richter D. (1987). The thymus as a neuroendocrine organ: Synthesis of vasopressin and oxytocin in human thymic epithelium. Ann. N.Y. Acad. Sci. **496:** 56–66.

Geenen V., Legros J.J., Hazee-Hagelstein M.T., Louis-Kohn F., Lecomte-Yerna M.J., Demoulin A., and Franchimont, P. (1985). Release of immunoreactive oxytocin and neurophysin I by cultured luteinizing bovine granulosa cells. Acta Endocrinol. (Copenhagen) **110:** 263–270.

Geenen V., Robert F., Fatemi M., Defresne M.P., Boniver J., Legros J.J., and Franchimont P. (1988b). Vasopressin and oxytocin: Thymic signals and receptors in T cell ontogeny. *In:* Recent progress in posterior pituitary hormones, Yoshida S. and Share L. Eds. (New York: Elsevier), pp. 303–310.

Geenen V., Martens H., Robert F., Legros J.J., Defresne M.P., Boniver J., Martial J. Lefèbvre P.J. and Franchimont P. (1991a). Thymic cryptocrine signaling and the immune recognition of self neuroendocrine functions. Prog. NeuroendocrinImmunol. **4:** 135–142.

Geenen V., Robert F., Martens H., Benhida A., De Giovanni G., Defresne M.P., Boniver J., Legros J.J., Martial J., and Franchimont P. (1991a). Biosynthesis and paracrine /cryptocrine actions of neurohypophysial-related peptides in the thymus. Mol. Cell. Endocrinol. **76:** C27–C31.

Geppetti P., Frilli S., Renzi D., Santicioli P., Maggi C.A., Theodorsson E., and Fanciullacci M. (1989). Distribution of calcitonin gene-related peptide-like immunoreactivity in various rat tissues: Correlation with substance P and other tachykinins and sensitivity to capsaicin. Regul. Pept. **23:** 289–298.

Geppetti P., Maggi C.A., Zecchi-Orlandini S., Santicioli P., Meli A., Frilli S., Spillantini M.G., and Amenta F. (1987). Substance P-like immunoreactivity in capsaicin-sensitive structures of the rat thymus. Regul. Pept. **18:** 312–329.

Geppetti P., Theodorsson-Norheim E., Ballerini G., Alessandri M., Maggi C.A., Santicioli P., Amenta F., and Fanciullacci M. (1988). Capsaicin-sensitive tachykinin-like immunoreactivity in the thymus of rats and guinea-pigs. J. Neuroimmunol. **19:** 3–9.

Giraud F., Fabien N., Auger C., Girod C., Loire R., and Monier J.C. (1990). Human epithelial thymic tumours: Heterogeneity in immunostaining of epithelial cell markers and thymic hormones. Thymus **15:** 15–29.

Gomariz R.P., Lorenzo M.J., Cacicedo L., Licente A., and Zapata A.G. (1990). Demonstration of immunoreactive vasoactive intestinal peptide (IR-VIP) and somatostatin (IR-SOM) in rat thymus. Brain Behav. Immun. **4:** 151–161.

Haynes B.F., Shimizu K., and Eisenbarth G.S. (1983). Identification of human and rodent thymic epithelium using tetanus toxin and monoclonal antibody A2B5. J. Clin. Invest. **71:** 9–14.

Herbst W.M., Kummer W., Hofmann W., Otto H., and Heym C. (1987). Carcinoid tumors of the thymus. An immunohistochemical study. Cancer **60:** 2465–2470.

Hunt N.H., Perris A.D., and Sandford P.A. (1977). Role of vasopressin in the mitotic response of rat bone marrow cells to haemorrhage. J. Endocrinol. **72:** 5–16.

Ivell R., and Richter D. (1984). Structure and comparison of the oxytocin and vasopressin genes from the rat. Proc. Natl. Acad. Sci. USA **81:** 2006–2010.

Jard S., Gaillard R.C., Guillon G., Marie J., Schoenenberg P., Muller A.F., Manning M., and Sawyer W.H. (1986). Vasopressin antagonists allow demonstration of a novel type of vasopressin receptor in the rat adenohypophysis. Molec. Pharmacol. **30:** 171–177.

Jevremovic M., Barbijeri M., Kovacevic D., Arambasic M., Kartaljevic G., Natalic D.J., and Pazin S. (1990). Identification of neuroendocrine oxytocic activity of the human fetal thymus. Thymus **15:** 181–185.

Kurihara M., Katamine S., and Saavedra J.M. (1987). Atrial natriuretic peptide, ANP (99-126), receptors in rat thymocytes and spleen cells. Biochem. Biophys. Res. Commun. **145:** 789–796.

Land H., Grez M., Ruppert S., Schmale H., Rehbein N., Richter D., and Schuetz G. (1983). Deduced amino acid sequence from the bovine oxytocin-neurophysin I precursor cDNA. Nature **302:** 342–344.

Land H., Schuetz G., Schmale H., and Richter D. (1982). Nucleotide sequence of cloned cDNA encoding bovine arginine vasopressin-neurophysin II precursor. Nature **295:** 299–303.

Le P.T., Denning S.M., Haynes B.F., and Singer K.H. (1990). Thymic epithelial-cell-derived cytokines and their roles in thymic precursor cell differentiation. Res. Immunol. **141:** 271–275.

Legros J.J. (1975). The radioimmunoassay of human neurophysins: Contribution to the understanding of the physiopathology of neurohypophyseal function. Ann. N.Y. Acad. Sci. **248:** 281–303.

Legros J.J., and Ansseau M. (1989). Increased basal plasma vasopressin-neurophysin in mania. Horm. Res. **31:** 55–58.

Legros J.J., Louis F., Demoulin A., and Franchimont P. (1976). Immunoreactive neurophysins and vasotocin in human foetal pineal glands. J. Endocrinol. **69:** 289–294.

Lorenz R.G., and Allen P.M. (1989). Thymic cortical epithelial cells can present self-antigens in vivo. Nature **337:** 560–562.

Lorton D., Bellinger D.L., Felten S.Y., and Felten, D.L. (1990). Substance P innervation of the rat thymus. Peptides **11:** 1269–1275.

Maggi M., Malowzowski S., Kassis S., Guardabasso V., and Rodbard D. (1987). Identification and characterization of two classes of receptors for oxytocin and vasopressin in porcine tunica albuginea, epididymis, and vas deferens. Endocrinol. **120:** 986–994.

Marchetti B., Guarcello V., Morale M.C., Bartoloni G., Farinella Z., Cordaro S., and Scapagnini U. (1989). Luteinizing hormone-releasing hormone-binding sites in the rat thymus: Characteristics and biological function. Endocrinology **125:** 1025–1036.

Markwick A.J., Lolait S.J., and Funder J.W. (1986). Immunoreactive arginine vasopressin in the rat thymus. Endocrinology **119:** 1690–1696.

Marrack P., McCormack J., and Kappler J. (1989). Presentation of antigen, foreign major histocompatibility complex proteins and self by thymus cortical epithelium. Nature **338:** 503–505.

Martens H., Robert F.R., Legros J.J., Geenen V., and Franchimont P. (1992). Expression of functional neurohypophysial peptide receptors by murine immature and cytotoxic T cell lines. Prog. NeuroEndocrinImmunol., in press.

Michell R.H., Kirk C.J. and Billah M.M. (1979). Hormonal stimulation of phosphatidylinositol breakdown, with particular reference to the hepatic effects of vasopressin. Biochem. Soc. Trans. **7:** 861–865.

Morley S.D., Schönrock C., Heierhorst J., Figueroa J., Lederis K., and Richter D. (1990). Vasotocin genes of the teleost fish *Catostomus commersoni:* Gene structure, exon-intron boundary, and hormone precursor organization. Biochemistry **29:** 2506–2511.

Nicolas J.F., Reano A.; Kaiserlian D., and Thivolet J. (1989). Epithelial cell heterogeneity in mammalian thymus: Monoclonal antibody to high molecular weight keratins exclusively binds to Hassall's corpuscles. Histochem. J. **21:** 357–364.

Nieuwenhuis J.J. (1984). Arginine vasotocin (AVT), an alleged hormone of the mammalian pineal gland. Life Sci. **35:** 1713–1724.

Pavel S. (1975). Vasotocin biosynthesis by neurohypophysial cells from human fetuses. Evidence for its ependydimal origin. Neuroendocrinol. **19:** 150–156.

Pavel S., Goldstein R., and Calb M. (1975). Vasotocin content in the pineal gland of foetal, newborn and adult male rats. J. Endocrinol. **66:** 283–289.

Piantelli M., Maggiano N., Larocca L.M., Ricci R., Ranelletti F.O., Lauriola L., and Capelli A. (1990). Neuropeptide-immunoreactive cells in human thymus. Brain Behav. Immun. **4:** 189–197.

Ransom J., Wu R., Fischer M., and Zlotnik A. (1991). Antigen presenting ability of thymic macrophages and epithelial cells: evidence for defects in the antigen processing function of thymic epithelial cells. Cell. Immunol. **134:** 180–190.

Richter, D., and Schmale H. (1984). Vasopressin expression in normal and diabetes insipidus (Brattleboro) rats. Trends Neurosci. **7:** 317–319.

Robert F., Geenen V., Schoenen J., Burgeon E., De Groote D., Defresne M.P., Legros J.J. and Franchimont P. (1991). Colocalization of immunoreactive oxytocin, vasopressin and interleukin-1 in human thymic epithelial neuroendocrine cells. Brain, Behavior and Immunity **5:** 102–115.

Robert F., Léon-Henri B.P., Chapleur-Chateau M.M., Girr M.N., and Burlet A.J. (1985). Comparison of three immunoassays in the screening and characterization of monoclonal antibodies against arginine-vasopressin. J. Neuroimmunol. **9:** 205–220.

Rozengurt E. (1986). Early signals in the mitogenic response. Science **234:** 161–166.

Savino W., and Dardenne M. (1984). Thymic hormone-containing cells VI. Immunohistologic evidence for simultaneous presence of thymulin, thymopoietin and thymosin $\alpha_1$ in normal and pathological human thymuses. Eur. J. Immunol. **14:** 987–991.

Scherbaum W.A., and Bottazzo G.F. (1983). Autoantibodies to vasopressin cells in idiopathic diabetes insipidus: Evidence for an autoimmune variant. Lancet **1:** 897–901.

Shigematsu K., Saavedra J.M., and Kurihara M. (1986). Specific substance P binding sites in rat thymus and spleen: In vitro autoradiographic study. Regul. Pept. **16:** 147–156.

Smith A., and McIntosh N. (1983). Neurohypophyseal peptides in the human fetus: Presence in pituitary extracts of immunoreactive arginine vasotocin. J. Endocrinol. **99:** 441–445.

Smitz S., Legros J.-J., Franchimont P., and Le Maire M. (1988). Identification of vasopressin-like peptides in the plasma of a patient with the syndrome of inappropriate secretion of antidiuretic hormone and oat cell carcinoma. Acta Endocrinol. (Copenhagen) **119:** 567–574.

Söder O., and Hellström P.M. (1989). The tachykinins neurokinin A and physalaemin stimulate murine thymocyte proliferation. Int. Arch. Allergy Appl. Immunol. **90:** 91–96.

Sundler F., Carraway R.E., Hakanson R., Alumets J., and Dubois M.P. (1978). Immunoreactive neurotensin and somatostatin in the chicken thymus. Cell Tiss. Res. **194:** 367–376.

Vollmar A.M., and Schulz R. (1990). Atrial natriuretic peptide is synthesized in the human thymus. Endocrinology **126:** 2277–2280.

Von Gaudecker B., Steinmann G.G., Hansmann M.L., Harpprecht J., Milicevic N.M., and Muller-Hermelink H.K. (1986). Immunohistochemical characterization of the thymic microenvironment. A light-microscopic and ultrastructural immunocytochemical study. Cell Tissue Res. **244:** 403–412.

Webb S.R., and Sprent J. (1990). Tolerogenicity of thymic epithelium. Eur. J. Immunol. **20:** 2525–2528.

Weihe E., Muller S., Fink T., and Zentel H.J. (1989). Tachykinins, calcitonin gene-related peptide and neuropeptide Y in nerves of the mammalian thymus: Interactions with mast cells in autonomic and sensory neuroimmunomodulation? Neurosci. Lett. **100:** 77–82.

Wekerle H., and Ketelsen, U.P. (1980). Thymic nurse cells. Ia-bearing epithelium involved in T-lymphocyte differentiation? Nature **283:** 402–404.

Whitfield J.F., MacManus J.P., and Gillan D.J. (1970). The possible mediation by cyclic AMP of the stimulation of thymocyte proliferation by vasopressin and the inhibition of this mitogenic action by thyrocalcitonin. J. Cell Physiol. **76:** 65–76.

